# Supplementary material for: Parasitic capacitance modeling and measurements of conductive yarns for e-textile devices
Source: Nat Commun. 2023 May 15;14:2785. doi: 10.1038/s41467-023-38319-6 (PMC10185586; doi:10.1038/s41467-023-38319-6)
Supplement: Supplementary file 1 — Supplementary Information [file 41467_2023_38319_MOESM1_ESM.pdf]

Supplementary Information

Parasitic Capacitance Modeling and Measurements of Conductive Yarns for e-Textile Devices

Ziqi Qu[1,2], Zhechen Zhu[1,3], Yulong Liu[1,4], Mengxia Yu[1,5], Terry Tao Ye[1,]*

[1]Department of Electrical and Electronic Engineering, Southern University of Science and Technology, Shenzhen, 518055, China

[2]Department of Nanotechnology, University of Pennsylvania, Philadelphia, Pennsylvania, 19104, U.S.

[3]Department of Electrical Engineering, University of Pennsylvania, Philadelphia, Pennsylvania, 19104, U.S.

[4]Department of Applied Physics, The Hong Kong Polytechnic University, Hong Kong.

[5]Department of Electrical and Computer Engineering, National University of Singapore, Singapore.

[*]*Corresponding Author, email: yet@sustech.edu.cn*

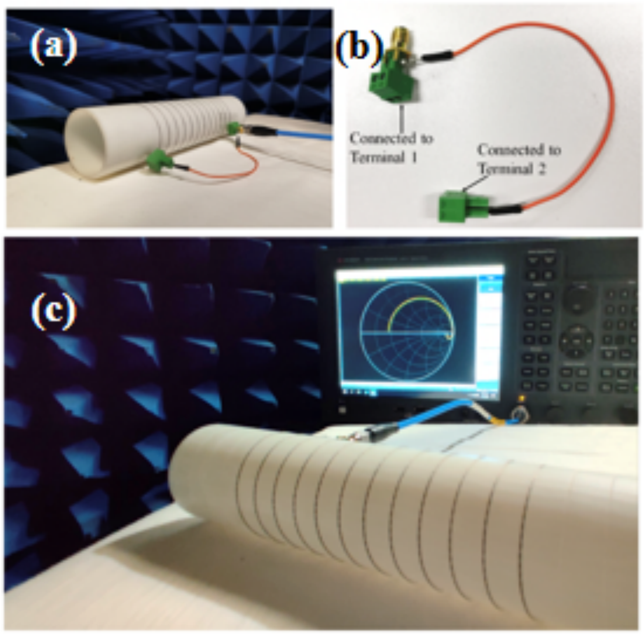

Supplementary Fig. 1. Setup for Helical Inductor Measurement **(a)** Helical inductor structure, **(b)** Port extension, **(c)** Measurement setup with a VNA

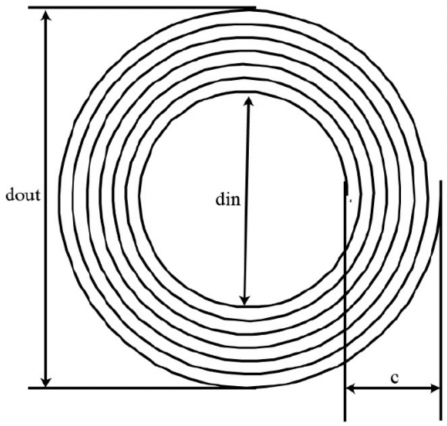

Supplementary Fig. 2. Planar spiral coil inductor and parameters for inductance estimation.

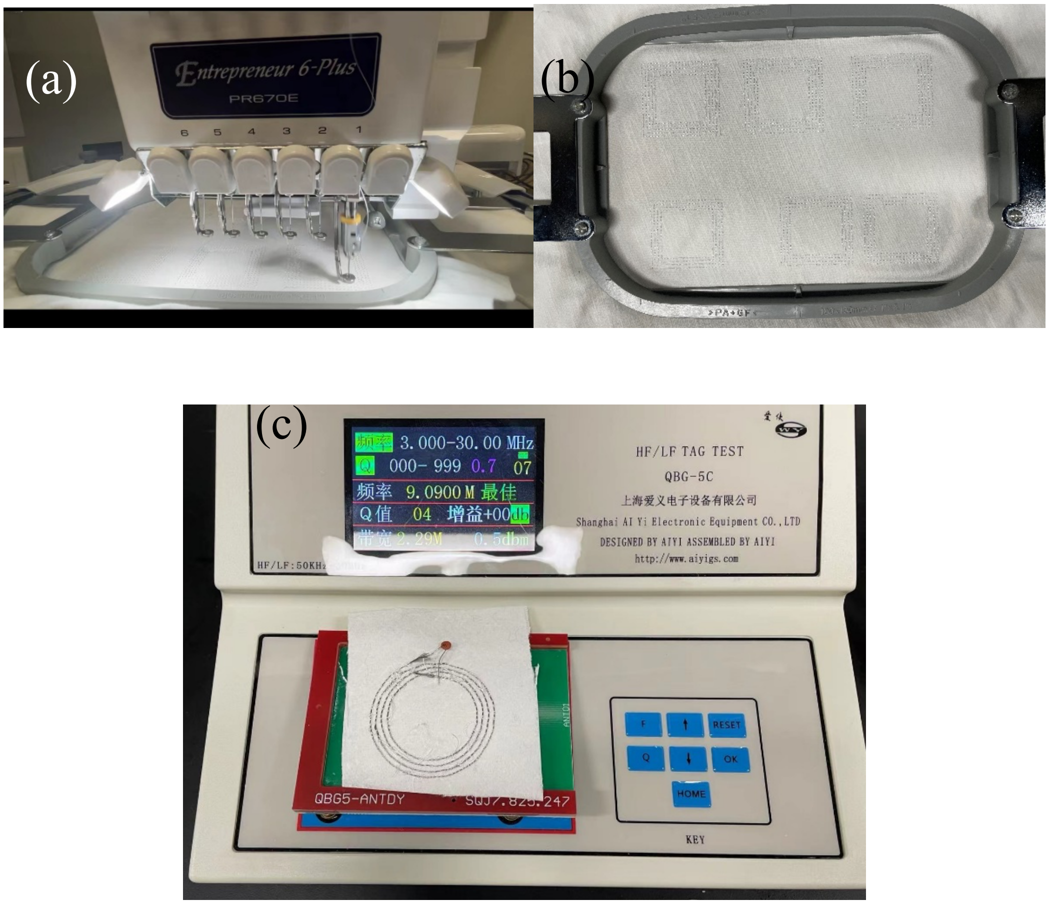

Supplementary Fig. 3. **E**mbroidered spiral coils fabrication and measurement setup **(a)** Embroidery machine **(b)** Embroidered spiral coil inductors **(c)** Measurement setup using a HF/LF RFID testing equipment

Supplementary Table 1. The GA Rules Specification.

**Stop Criteria**	**'MaxGenerations' (400)**
**Selection**	'selectionstochunif'
**Crossover**	0.8
**Mutation Rate**	0.01

Supplementary Table 2. Solution Vector Initialization and Variables Constraints.

Variables	Stage I initial value	Constraints	Stage II initial value	Constraints
$\boldsymbol{R}_{\mathbf{i}}$	$\frac{R_{\mathrm{t}}^{\mathrm{copper}}}{N}$	[1, 20]	$\frac{R_{\mathrm{t}}^{\mathrm{yarn}}}{N}$	[1, 20]
$\boldsymbol{L}_{\mathbf{i}}$	$\frac{L_{\mathrm{t}}^{\mathrm{copper}}}{N}$	[1e-7, 1e-5]	$\frac{L_{\mathrm{t}}^{\mathrm{yarn}}}{N}$	[1e-7, 1e-5]
$\boldsymbol{C}_{\mathbf{s}}$	C_{s}	[1e-13, 1e-10]	$C_{\mathrm{s}}^{\mathrm{copper}}$	fixed
$\boldsymbol{C}_{\mathbf{c}}$	C_{c}	[1e-15, 1e-12]	$C_{\mathrm{c}}^{\mathrm{copper}}$	fixed
$\boldsymbol{C}_{\mathbf{p}}$	0	fixed	C_{p}	[1e-14, 1e-11]

Supplementary Table 3. Parameters of the DUT Supporter.

Parameter	Value (mm)
**Supporter Radius $\boldsymbol{r}$**	22
**Supporter Total Length $\boldsymbol{l}$**	205
**Pitch Between Turns $\boldsymbol{p}$**	10
**Width of Groove $\boldsymbol{\delta}$ pre-carved on ABS cylinder**	0.5

Supplementary Table 4. Design Parameters of the Embroidered Planar Spiral Coils.

Number of Turns	Space between Turns, S (mm)	Inner Diameter, d_{in} (mm)	Outer Diameter, d_{out} (mm)
5	2	32	51.0
6	2	32	55.6
7	2	32	60.2
8	2	32	64.8

Supplementary Table 5. Comparison of Theoretical and Measured Resonant Frequencies of the Planar Spiral Coils Resonator with Different Turn Numbers (Embroidered with AMBERSTRAND® 166 Yarns, Connected to a 25 pF Capacitor).

Number of Turns	Calculated Inductance (μH)	Calculated Frequency (MHz)	Measured Frequency (MHz)	Deviation (MHz)
**5**	1.567	25.431	25.205	-0.226
**6**	2.256	21.192	21.067	-0.125
**7**	3.019	18.320	18.131	-0.189
**8**	3.946	16.025	16.009	-0.016
**Average**	-	-	-	-0.139

Note: The measured frequency is the mean value of 5 repeated measurements.

Supplementary Table 6. Revised Resonant Frequency Incorporating Parasitic Capacitances (AMBERSTRAND® 166 Yarn, Connected to a 25 pF Capacitor).

Number of Turns	Total Length (cm)	Yarn Parasitic Capacitance (pF)	Amended Frequency (MHz)	Measured Frequency (MHz)	Deviation (MHz)
**5**	61.26	0.216	25.319	25.205	-0.114
**6**	77.28	0.273	21.078	21.067	-0.011
**7**	94.56	0.334	18.199	18.151	-0.048
**8**	113.10	0.399	15.898	16.009	0.111
**Average**	-	-	-	-	-0.015

Note: The measured frequency is the mean value of 5 repeated measurements.
